# Self-Medication Behaviors: Determinants, Motivations, and Safety Practices Among Health Sciences Students

**DOI:** 10.3390/healthcare14131910

**Published:** 2026-07-01

**Authors:** Dominik Olejniczak, Magdalena Łopatek, Agnieszka Wasiluk, Katarzyna Domosławska-Żylińska, Maria Piotrowicz, Aleksandra Kielan, Urszula Mazur, Anna Staniszewska

**Affiliations:** 1 Department of Health Promotion and Public Health System Implementation, National Institute of Public Health NIH—National Research Institute, 24 Chocimska St., 00-791 Warsaw, Poland; dominik.olejniczak@wum.edu.pl (D.O.);; 2 Division of Public Health, Faculty of Health Science, Medical University of Warsaw, 61 Żwirki i Wigury St., 02-091 Warsaw, Poland; 3 Department of Human Physiology and Pathophysiology, University of Warmia and Mazury in Olsztyn, 30 Warszawska St., 10-082 Olsztyn, Poland; 4 Department of Experimental and Clinical Pharmacology, Medical University of Warsaw, 61 Żwirki i Wigury St., 02-091 Warsaw, Poland

**Keywords:** self-medication, OTC medicines, health behavior, health literacy, health sciences students

## Abstract

**Background/Objectives**: Self-medication (SM) is a global public health phenomenon with both benefits and risks. Evidence on its determinants remains inconsistent, particularly among university students, and limited in Central and Eastern Europe despite high OTC availability and variable health literacy. This study aimed to assess SM behaviors among health sciences students and identify sociodemographic and health-related determinants of OTC use. **Methods**: The cross-sectional CAWI survey was conducted between November 2024 and March 2025 among 435 students of the Medical University of Warsaw selected through purposive sampling. Univariable and multivariable logistic regression analyses were performed in the study population to characterize respondents practicing SM. Detailed analyses of SM behaviors included only respondents reporting self-medication (n = 278; 64.2%). Associations between sociodemographic and health-related characteristics and selected SM behaviors were assessed using the chi-square (χ^2^) test, with false discovery rate correction applied using the Benjamini–Hochberg procedure. Questionnaire reliability was confirmed using Cohen’s kappa coefficients ranging from 0.63 to 1.00. Statistical significance was set at *p* < 0.05. **Results**: The most commonly used medicines were analgesics (97.5%), vitamins (54.7%), and antipyretics (48.2%). Adjusted multivariable analysis showed that the odds of SM were higher among women (AOR = 4.11, 95% CI = 2.11–8.02), Emergency Medical Services students (AOR = 3.57, 95% CI = 1.45–8.75), master’s degree students (AOR = 3.45, 95% CI = 1.96–5.88), and students living in large cities with more than 500,000 inhabitants (AOR = 2.63, 95% CI = 1.43–5.00). Motivations, perceived benefits, risks, and adherence to package leaflet instructions differed significantly by respondent characteristics. **Conclusions**: SM behaviors are influenced by sociodemographic and health-related factors. Targeted education on rational OTC use, professional responsibility, and critical appraisal of health information is needed. Safe SM practices among health sciences students may support their future role in promoting responsible self-medication.

## 1. Introduction

Self-medication has become an integral component of contemporary health-related behavior and is increasingly recognized as an important public health issue. While the use of non-prescription medicines may facilitate timely management of minor ailments and reduce unnecessary healthcare utilization, inappropriate medication use may result in adverse drug reactions, drug–drug interactions, masking of serious diseases, and delays in seeking professional medical advice [1,2]. Consequently, self-medication should be considered not only an individual therapeutic choice but also a behavior with potential implications for population health. The growing popularity of self-medication has been observed worldwide, although its prevalence varies considerably across countries and regions. Such variation reflects differences in healthcare accessibility, medicine regulations, socioeconomic conditions, cultural attitudes towards self-care, and levels of health literacy [3,4]. In the European Union, approximately one-third of adults report engaging in self-medication practices, whereas studies conducted in Poland indicate substantially higher prevalence estimates, ranging from 59% to 89% [2,5]. This phenomenon has been further accelerated by the COVID-19 pandemic, which permanently altered patient behaviors, increased fear of infection in medical facilities, and forced a shift toward systemic self-reliance [6]. The widespread use of over-the-counter (OTC) medicines is facilitated by their broad availability in non-pharmacy outlets (such as supermarkets and gas stations), intensive marketing, and public perception that non-prescription medicines are inherently safe [2].

The decision to self-medicate is influenced by a complex interplay of individual and systemic factors. Previous positive experiences with OTC medicines, financial barriers, long waiting times for medical appointments, and dissatisfaction with healthcare services have all been associated with a greater likelihood of self-medication [2]. In addition, the rapid expansion of digital health resources has transformed the way individuals obtain information about symptoms and treatments. Online search engines, health-related websites, mobile applications, and social media platforms increasingly serve as sources of therapeutic guidance, potentially affecting perceptions of medicine safety and influencing treatment decisions without professional consultation [7,8,9,10,11,12,13]. This digital shift often leads to “cyberchondria,” where excessive online symptom searching amplifies anxiety and drives individuals toward unverified self-treatment [14].

Young adults constitute a population particularly prone to self-medication. The transition from adolescence to independent adulthood is accompanied by increasing responsibility for personal health decisions, often combined with limited experience in navigating healthcare systems. University students frequently encounter academic pressure, time constraints, and irregular healthcare-seeking behaviors, which are factors that may encourage reliance on self-care strategies, including the use of OTC medicines. Recent evidence indicates that self-medication is highly prevalent among university students worldwide, with prevalence estimates in many countries exceeding those observed in the general population. These findings have highlighted the need for educational interventions aimed at improving medication literacy and promoting responsible medicine use [15]. Students enrolled in health sciences programs represent a particularly relevant subgroup for investigation. Their education provides them with greater exposure to pharmacological knowledge and health-related information than their non-medical peers. Nevertheless, increased theoretical knowledge does not necessarily translate into safer medication practices, and several studies have demonstrated that inappropriate self-medication remains common even among future healthcare professionals [16]. This disparity, often described as a gap between theoretical awareness and actual behavior, is frequently driven by overconfidence in one’s ability to self-diagnose [17,18]. Understanding their attitudes and behaviors is especially important because their personal experiences and beliefs regarding medicine use may influence future patient counseling and professional practice. Despite the growing body of international literature, evidence regarding self-medication among university students in Central and Eastern Europe remains limited. This region possesses unique systemic characteristics, including specific post-communist healthcare transformations, high out-of-pocket pharmaceutical spending, and a deeply rooted culture of pharmacy-based self-care [19,20]. Existing studies have reported heterogeneous findings, making it difficult to draw clear conclusions regarding the determinants and patterns of self-medication in this region [16]. Therefore, the present study aimed to assess self-medication behaviors among health sciences students and to examine selected sociodemographic and health-related factors associated with decision-making related to the use of OTC medicines.

## 2. Materials and Methods

The World Health Organization (WHO) defines SM as the practice of using medicinal products to treat self-diagnosed disorders or symptoms, as well as the periodic or regular use of previously prescribed medicines for chronic or recurrent conditions or symptoms [21]. Although the WHO definition encompasses several forms of self-directed medication use, including the continued use of previously prescribed medicines, the present study focuses specifically on the use of over-the-counter (OTC) medicines without prior medical consultation. This operational definition was adopted because OTC medicines represent the most common and legally accessible form of self-medication among university students and have been the primary focus of most epidemiological studies investigating self-medication behaviors in this population.

The survey was designed as a cross-sectional survey and was conducted from November 2024 to March 2025, using the CAWI (Computer-Assisted Web Interviewing) method. This approach was selected because it enabled efficient access to a large group of students, who commonly use online tools in academic communication. Participants were recruited through purposive sampling, as the study focused on a clearly defined population of students enrolled in health-related fields, whose knowledge and behaviors regarding the use of over-the-counter medications constituted the main subject of the analysis.

### 2.1. Participants

The study included undergraduate and graduate students (1st–5th year, full-time and part-time) of the Faculty of Health Sciences at the Medical University of Warsaw, Poland. An invitation to participate in the study, together with a link to the survey, was sent to students by e-mail through the university’s institutional communication channels.

Participants were informed about the scientific nature of the study, the voluntary nature of participation, and their right to withdraw at any stage while completing the survey. All data were stored in a manner ensuring full confidentiality and security. Access to the data was restricted solely to the authors of the study, and no information enabling the identification of respondents was collected or shared with third parties. The study received a positive opinion from the Bioethics Committee at the Medical University of Warsaw (Approval No. AKBE/306/2024, dated 18 November 2024).

Eligible participants were students of the Faculty of Health Sciences at the Medical University of Warsaw who had active student status during the data collection period, were enrolled in one of the fields of study included in the analysis, were at least 18 years of age, and provided informed consent to participate in the study. Individuals who did not meet the inclusion criteria, did not provide consent to participate, or did not complete the questionnaire in full were excluded from the analyses. These criteria were adopted to ensure that the study population corresponded to the target group of the research and that the analyses were based only on complete and comparable survey data.

Out of 2001 enrolled students, 435 completed the questionnaire, corresponding to a response rate of 21.7%. The relatively low response rate may have resulted from the voluntary nature of participation, limited interest in the study topic, failure to open the invitation e-mail, limited time availability among students, or the message being overlooked in daily academic correspondence. The possibility of selection bias should also be taken into account, as students who were more interested in self-medication or had personal experience with the use of over-the-counter medications may have been more likely to participate in the study.

**Figure 1:**
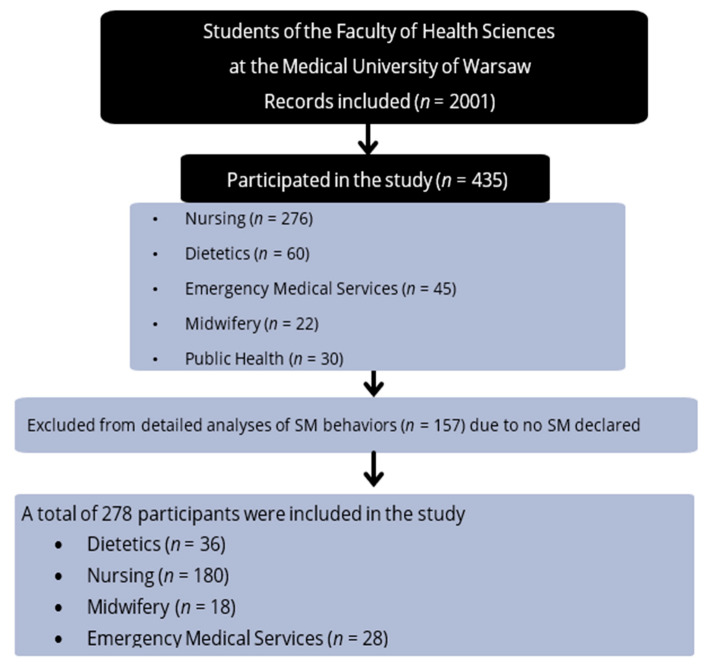
Study sample selection process.

A total of 435 students were included in the study. In the first stage, it was assessed whether respondents practiced self-medication, defined as the use of over-the-counter medicines without medical consultation. For students who did not report self-medication, participation in this part of the analysis ended at this stage. Detailed questions on self-medication behaviors were addressed only to those who declared such practices. These analyses included 278 students from the following fields of study: Nursing (n = 180), Dietetics (n = 36), Emergency Medical Services (n = 28), Midwifery (n = 18), and Public Health (n = 16) (Figure 1). Self-medication behaviors were analyzed in three areas: attitudes toward self-medication, behaviors related to purchasing and using over-the-counter medicines, and safety-related practices.

### 2.2. Questionnaire Design

Data were collected using a 19-item author-designed questionnaire covering two domains: sociodemographic information (8 questions), including health status, and self-medication behaviors (11 questions) (File S1). The SM section included six single-choice and five multiple-choice questions. The questionnaire was developed by the authors specifically for the purposes of this study, based on the study objective, the scope of the variables analyzed, and a review of the literature on self-medication and the use of over-the-counter medications. It was not adapted from a single standardized instrument. The questions were designed to cover the declaration of self-medication, attitudes towards self-medication, behaviors related to the purchase and use of over-the-counter medications, and safety-related practices concerning their use.

Before the main study was conducted, the instrument was assessed for face and content validity. Face validity was evaluated by analyzing the clarity, unambiguity, and acceptability of the questions among students participating in the pilot study. Content validity was assessed by experts in public health, who verified whether the items adequately reflected the main domains of interest, including self-medication practices, the use of over-the-counter medications, and safety-related behaviors.

The comprehensibility and acceptability analysis was conducted among 67 students. Mean completion time was 5 ± 2 min (ranging from 2 to 10 min). All students reported that the questionnaire format was good, the font size was large enough, and the questions were understandable; they also stated that they had no questions they did not want to answer. In respondents’ opinion, the questionnaire was sufficiently long (except for two students). 93% of students did not indicate difficulty in clearly answering the questions. During the survey, three respondents (4%) noted that OTC medicines could impact their health, the duration of use, and the potential consequences.

Reliability was assessed in a subgroup of 55 students using a two-week test–retest procedure, which included a comparison of two sets of responses provided by the same students. Test–retest reliability coefficients ranged from 0.63 to 1.00 over a 2-week interval (except for the question “Do you follow the information in the leaflet?”, which showed moderate agreement with a reliability coefficient of 0.51).

Cohen’s Kappa coefficient was used to assess the agreement of two measurements of a variable. For multiple-choice questions, each response option was coded as a separate dichotomous variable, where 0 indicated that the response option was not selected and 1 indicated that it was selected. The criteria for compliance, according to Landis and Koch, were as follows: 0–0.20 indicates slight agreement, 0.21–0.40 indicates fair agreement, 0.41–0.60 indicates moderate agreement, 0.61–0.80 indicates substantial agreement, and 0.81–1.00 indicates almost perfect agreement.

### 2.3. Statistical Analysis

Two analyses were carried out in this study. In the first part, univariable and multivariable logistic regression analyses were performed in the entire study population to characterize individuals practicing SM with respect to sociodemographic and health-related status. Respondents who selected “other” in the sex variable were excluded from this analysis; therefore, the sample size in the logistic regression analysis was N = 433. The dependent variable was SM (yes/no), coded as a binary variable (0/1), while independent variables (e.g., age, sex, field of study, place of residence, level of study, financial situation, and health status) were categorical. The selection of independent variables was based on the study objectives, the structure of the questionnaire, and previous literature indicating that sociodemographic and health-related characteristics may be associated with self-medication behaviors [15,22]. Crude odds ratios (COR) with 95% confidence intervals (CI) were calculated to determine the strength and direction of associations. Adjusted odds ratios (AOR) with 95% confidence intervals were calculated in the multivariable logistic regression model. All selected sociodemographic and health-related variables were entered simultaneously into the model to account for potential confounding. The multivariable model was estimated using the forced-entry method, with all selected predictors entered into the model in a single step. The reference categories were as follows: age > 31 years, male gender, Nursing, Master’s degree students, city >500,000 inhabitants, poor/very poor financial situation, poor/very poor self-assessed health status, and no chronic condition. Model fit was assessed using the model chi-square test, −2 log likelihood, Cox and Snell R^2^, Nagelkerke R^2^, the Hosmer–Lemeshow goodness-of-fit test, and classification accuracy. The final model was statistically significant, χ^2^(20) = 75.440, *p* < 0.001, with −2 log likelihood = 489.399, Cox and Snell R^2^ = 0.160, and Nagelkerke R^2^ = 0.219. The Hosmer–Lemeshow test indicated an acceptable model fit, χ^2^(8) = 6.649, *p* = 0.575, and the overall classification accuracy was 69.5%. Multicollinearity among the independent variables in the multivariable logistic regression model was assessed using tolerance values and variance inflation factors (VIFs). All VIFs were below 2, with a maximum value of 1.274, and tolerance values were ≥0.785, indicating no significant multicollinearity.

In the second part, analyses concerning SM-related behaviors included only respondents who declared using self-medication. Categorical variables were analyzed using the chi-square (χ^2^) test to assess associations between sociodemographic and health-related characteristics and selected self-medication behaviors. The false discovery rate was controlled using the Benjamini–Hochberg procedure. Respondents who indicated “other” in the gender variable were excluded due to the very small size of this subgroup.

The analyzed SM behaviors were grouped into three areas: (I) attitudes toward self-medication, (II) behaviors related to purchasing and using OTC medicines, and (III) safety-related practices. A total of 312 tests were analyzed. 84 were nominally significant, but after FDR correction, 27 remained significant. For multiple-choice questions, each response option was coded as a separate dichotomous variable, where 0 indicated that the option was not selected and 1 indicated that it was selected. Therefore, each response option was analyzed as an independent binary.

All statistical analyses were performed at a significance level of *p* < 0.05 using Statistica software, version 13 (StatSoft Polska, Kraków, Poland).

## 3. Results

### 3.1. Characteristics of Respondents (N = 435) and Sociodemographic and Health Status Factors Associated with Undertaking Self-Medication

The sociodemographic and health-related characteristics of the respondents are presented in Table 1. The mean age was 23 years (SD = 4.2), with an age range from 18 to 45 years. The majority of respondents were women (85.5%) and nursing students (63.9%). More than half of the full-time students who accounted for 95.0% of all respondents were enrolled in second-cycle (master’s) programs. In terms of place of residence, more than half of the respondents came from large cities with populations exceeding 500,000 inhabitants (52.0%). Most respondents assessed their financial situation as good (51.3%) or average (29.0%), with 17.2% rating it as very good. Regarding self-rated health status, most respondents assessed their health as good (60.9%) or very good (12.6%). Nearly one in three respondents (30.3%) reported having a chronic disease.

Self-medication, was reported by 278 respondents (64.2%), while 155 (35.8%) stated that they did not engage in this practice. In the multivariable analysis, female gender was associated with more than fourfold higher odds of self-medication compared with male gender. Students of Emergency Medical Services had higher odds of self-medication compared with Nursing students. In contrast, Bachelor’s degree students had lower odds of self-medication compared with Master’s degree students, and students living in towns with ≤100,000 inhabitants had lower odds compared with those living in cities with >500,000 inhabitants (*p* < 0.05). The remaining variables did not show a significant association with self-medication in the adjusted model. In the univariable analysis, self-assessed overall health was associated with self-medication; however, this association was not confirmed in the multivariable model (Table 2).

### 3.2. Self-Medication Behaviors Among Respondents Who Undertake Self-Medication/Using OTC Medicines (N = 278)

Analyses of SM behaviors focused on participants who reported OTC medicine use (Table 3). In the studied group, decisions to SM were mainly motivated by easy access to medicines (78.4%) and lack of time for a medical visit (50.7%). The most frequently indicated advantages of self-medication included saving time (36.3%) and a sense of engagement and self-determination in managing one’s own health (24.8%). Some respondents (13.3%) did not perceive any particular advantages of SM. Most participants identified the following risks: underestimation of symptoms of a serious diseases (68.7%), the possibility of incorrect self-diagnosis (57.2%).

Among the products used, analgesics (97.5%), vitamins (54.7%), and antipyretics (48.2%) were the most common. The primary place of purchase for OTC medicines was pharmacies (92.4%). The majority of respondents used such products for self-medication several times a year (61.2%). The duration of use typically ranged from 4 to 7 days (46.4%) or 2–3 days (37.8%). The main sources of information about OTC medicines were the Internet (66.6%) and pharmacists (57.2%), with nearly half of the respondents also relying on family members (47.5%) and physicians (47.1%).

Regarding safety-related behaviors, most users reported always or sometimes informing their physician about all products they were taking (79.9%). Most of them (89.2%) also declared that they always or almost always followed the instructions provided in the package leaflet, and they never used medicines past their expiration date (77.3%).

### 3.3. Sociodemographic and Health-Related Determinants of Selected Self-Medication Behaviors

The analyzed behaviors were grouped into three areas: (I) attitudes toward self-medication, (II) behaviors related to purchasing and using OTC medicines, and (III) safety-related practices. Among the variables analyzed within these categories, almost all of them showed a statistically significant association with the respondents’ sociodemographic factors and health status.

#### 3.3.1. Attitudes Toward SM

The analysis was carried out to assess associations between sociodemographic and health-related characteristics of respondents and selected self-medication behaviors from the area of attitudes (Table 4).

The reasons for self-medication reported by respondents varied depending on age, field of study, place of residence, self-assessment of financial situation, and presence of chronic disease. Individuals who rated their financial situation as average more often pointed to limited financial resources for purchasing medicines as a reason for self-medicating. Dissatisfaction with medical services also differed according to respondents’ age and field of study. This reason was indicated most often by students aged over 31 years and by midwifery students. When it comes to health status, respondents with chronic conditions more frequently indicated dissatisfaction with medical services and limited financial resources for purchasing medicines as reasons for self-medication.

Regarding the perceived advantages of self-medication, respondents from smaller towns significantly more frequently indicated saving money.

In terms of respondents’ perceived risks associated with self-medication, women more frequently emphasized the risk of misdiagnosing ailments and the possibility of adverse drugs interactions. Men, on the other hand, more often pointed to the risk of downplaying disease symptoms, similarly to residents of large cities. This risk also differed by field of study and was indicated most often by Emergency Medical Services students, followed by Public Health and Dietetics students, whereas it was least frequently reported by Midwifery students. Respondents who rated their overall health as fair, significantly more often than other participants indicated the risk of developing dependence on OTC medicines and the possibility of drug interactions.

#### 3.3.2. Practices Related to the Purchase and Use of OTC Medicines

The analysis was carried out to assess associations between sociodemographic and health-related characteristics of respondents and selected self-medication behaviors from the area of purchase and use of OTC medicines (Table 5).

The surveyed OTC medicine users reported taking various types of these products, for different ailments and health needs.

The health-related purpose of purchase varied depending on gender, level of study, and the presence of chronic disease. Women more often than men used antispasmodic medicines. Master’s students more frequently reported using antipyretics, whereas Bachelor’s students more often declared using sedatives and sleep aids. Respondents with chronic conditions more often declared using analgesics. When it comes to self-rated overall health, it did not differentiate consumers’ behaviors with regard to this particular purpose of purchase.

With regard to sources of information about OTC medicines, differences were observed according to age, gender, field of study, level of study, place of residence, self-assessment of financial situation, and the presence of chronic disease.

Students aged 18–20 years more often used the Internet as a source of information about OTC medicines, whereas those aged 27–30 years more frequently consulted family members. Women more often than men obtained information from physicians. A similar tendency was observed among individuals with good or very good financial status, for whom the doctor was also a preferred source of information, while respondents with a moderate financial situation were less likely to consult a physician. Differences were also observed by field and level of study. Midwifery students more frequently consulted family members as a source of information about OTC medicines. Master’s students more often used the Internet, whereas Bachelor’s students more frequently relied on family members.

Individuals living in smaller towns and rural areas more frequently relied on the knowledge of friends and family. The health status of the respondents influenced the sources of information about OTC medicines they chose. Individuals with chronic conditions, compared with others, more frequently indicated pharmacists.

#### 3.3.3. Safety-Related Practices

The analysis was carried out to assess associations between sociodemographic and health-related characteristics of respondents and selected self-medication behaviors from the area of safety-related practices (Table 6).

Following the recommendations provided in the package leaflet of OTC medicines was noticeably more common, among residents of large cities compared with others. In turn, the use of OTC medicines beyond their expiration date differed according to age. Students aged 27–30 years and those over 31 years more often reported using medicines past their expiration date.

## 4. Discussion

Our study confirms the high prevalence of self-medication among university students, as also described in previous studies [15,23,24]. Analgesics were the most commonly used products, followed by vitamins and antipyretics, in line with earlier findings [25]. The reasons for self-medication among the students surveyed were related to easy availability of OTC medicines and organizational barriers to accessing medical services, such as lack of time or long waiting times for appointments. Consistent with prior reports, these practices mainly involved mild ailments considered manageable without professional care. Confidence in one’s ability to assess symptoms, prior experience with similar conditions, and the easy availability of OTC medicines all contributed to this choice, while a demanding academic schedule may have further reinforced the tendency toward self-medication by limiting opportunities to seek medical consultation [11].

The analysis showed that each examined population characteristic was associated with differences in at least one aspect of self-medication behaviors. With regard to gender, the analysis revealed that women were more than four times as likely as men to report using OTC medicines. However, they simultaneously demonstrated greater caution in their use. Women significantly more and were more likely to recognize potential risks associated with self-medication, such as the possibility of drug interactions. This finding may reflect well-documented gender differences in health-related behaviors: women tend to exhibit lower health-related risk-taking tendencies [26], greater health awareness, and a stronger orientation toward preventive practices [27]. These factors may contribute to a more careful review of package leaflets and medicine labels before use. An additional observation emerging from our data is that men were less likely than women to consult a physician as a source of information about OTC medicines. This tendency is consistent with broader evidence showing that men generally make less use of healthcare services [28]. Such patterns may reflect greater confidence in autonomous decision-making among male students and a lower perceived need for professional input. However, reduced reliance on medical consultation may increase the risk of inappropriate self-medication, underscoring the importance of addressing gender-specific behaviors in educational strategies promoting safe OTC use.

In our analysis, self-medication was significantly more common among residents of large cities. This finding is consistent with studies reporting higher self-medication rates in urban populations, often attributed to greater availability of pharmacies, easier access to OTC medicines, and a higher level of health-related information exposure [29]. In our study, students living in large cities more often adhered to recommendations provided in the package leaflet and were more likely to recognize potential risks associated with self-medication, such as underestimating symptoms of a serious disease. This may suggest that urban residence is not only associated with easier access to OTC medicines, but also with greater exposure to professional sources of information, including pharmacists.

However, the literature also points to a different mechanism, indicating that residents of rural areas may resort to self-medication because of longer distances to healthcare facilities, limited access to specialists, and potential costs associated with medical consultations [30]. Thus, the relationship between place of residence and self-medication may be context-dependent. In urban settings, self-medication may be driven mainly by easy access to OTC products and convenience, whereas in rural areas it may result more from barriers to accessing professional healthcare. This interpretation is consistent with our findings showing that students from smaller towns more often relied on informal sources of information, such as family and friends, whereas those living in large cities more frequently identified pharmacists as a source of information about OTC medicines.

Although the literature highlights that self-medication is particularly common among patients with chronic diseases [29], our study did not confirm this trend. This discrepancy may be explained by the characteristics of our study population, which consisted of university students—a group that is generally younger, healthier, and less likely to engage in routine pharmacotherapy than typical chronic condition cohorts described in the literature. However, respondents with chronic conditions more often indicated dissatisfaction with previous medical services as one of the reasons for self-medication. This may be related to their more frequent contact with healthcare providers due to the chronic illness, which increases their awareness of limitations within the healthcare system.

Self-medication was more common among master’s degree students than among bachelor’s degree students. This may be related to greater independence, older age, and higher confidence in making health-related decisions among students at a more advanced stage of education. A similar pattern was reported by Banda et al., who found that year of study was the only independent predictor of self-medication among medical students at Copperbelt University in Zambia, with fourth-year students being more likely to self-medicate [31]. This interpretation is also supported by the observed differences in sources of information: master’s degree students more often used the Internet to obtain information about OTC medicines, whereas bachelor’s degree students more frequently relied on family members. This may suggest that students at earlier stages of education are more likely to seek advice from close informal sources, while more advanced students may make health-related decisions more independently.

Self-medication currently operates within a reality of unrestricted access to information, which promotes greater patient autonomy in managing personal health. The study demonstrated that among all sources of information, the Internet was chosen more frequently (66.6%) than pharmacists (57.2%) or physicians (47.1%). However, findings from other studies do not always follow this trend. In some research, traditional sources of information—such as package leaflets, pharmacists, and physicians—were reported as primary references, with the Internet playing a secondary role [32].

The prominence of the Internet as a source of information underscores the importance of digital health literacy. Individuals should be able to critically assess the credibility of diverse online sources, as the reliability of medical information varies widely across official health websites, pharmacy platforms, social media, and commercial content. Strengthening these competencies is essential for supporting safe and informed self-care practices.

Decisions regarding self-medication among students were primarily motivated by easy accessibility (78.4%), a lack of time for a medical visit (50.7%), and the expectation of effective and quick treatment results (46%). In another study conducted among Polish students [33], an additional factor, particularly among medical students, was the belief that their own treatment methods were superior to those recommended by licensed physicians. An interesting observation from this research was the absence of a financial barrier, especially among medical students. This finding is consistent with the results of our study, which showed that the economic situation did not have a significant impact on either the purchase of additional, non-prescribed medicines or overall health-related behaviors. However, the consistency across studies suggests that these motivations may reflect broader patterns in student populations rather than context-specific determinants. It is also possible that the reported lack of financial constraints reflects students’ perception of OTC medicines as relatively low-cost and low-risk products. This may underestimate the cumulative financial and health implications of frequent self-medication. Therefore, these results should be interpreted with caution, as they may not fully capture the complexity of decision-making processes related to self-medication.

From the perspective of the healthcare system, it is essential to promote rational SM, ensure its safety and effectiveness. No system is capable of continuously meeting all the health needs of its citizens. Therefore, self-medication should be practiced rationally. A necessary condition, however, is that decisions are based on medical knowledge and current professional recommendations [7]. Self-medication that is not grounded in safety principles described in the literature may, in extreme cases, lead to overlooking symptoms of disease, the risk of medicines interactions, or delays in diagnosis [34]. In this context, the role of healthcare professionals becomes crucial, as their accessibility enables them to serve as the primary point of contact and support patients in making informed, safe decisions. In some European Union countries, systemic solutions have been introduced that encourage patients to consult a pharmacist before seeing a physician, which not only increases health awareness but also helps reduce the burden on primary healthcare [7]. Nurses, dietitians, and midwives may play a similar role [7].

An additional aspect highlighted in the literature is the need for a clear distinction between medicines, dietary supplements, medical devices, and foods for special medical purposes [35]. The study assumed that self-medication refers to the independent use of OTC medications. However, the products purchased by respondents (such as vitamins, sedatives/sleep aids, and memory/concentration enhancers) may have different legal statuses on the market, such as dietary supplements or medical devices. Consumers are not experts on this matter. When purchasing a product, consumers do so primarily for a specific health-related purpose. This confirms the necessity of utilizing the full potential of all healthcare professions in education. Such an approach may significantly contribute to increasing awareness regarding health-related choices in the context of self-use of OTC medicines, thereby reducing the potential risk of purchasing an inappropriate product [36]. This highlights both the importance of obtaining accurate information from healthcare professionals before buying medications and the need to enhance health literacy within society, which is crucial for making informed health-related decisions daily. The high proportion (92.4%) of students in our study indicating pharmacies as their primary place for purchasing medications confirms high awareness within this group regarding the need for professional health education, including that provided by pharmacists.

When interpreting cross-country comparisons, it is important to consider specific characteristics of the Polish healthcare context. Poland is marked by broad accessibility of OTC medicines, including availability outside pharmacies, and by the prominent role of community pharmacies as an easily accessible first point of contact for health advice [37]. At the same time, variable waiting times for medical consultations and a strong reliance on self-care may influence medication-seeking behaviors differently than in other healthcare systems [38]. The post-COVID period is also relevant, as the pandemic reshaped access to healthcare, expanded telemedicine use, and further increased the tendency to manage minor health problems independently [39].

Self-management of health is an essential condition for the proper functioning of society, as caring for oneself ultimately means caring for others. Therefore, the goal should not be to discourage self-medication, but to ensure that it is practiced as safely as possible [40].

This study has several limitations that should be considered when interpreting the findings. First, the sample consisted exclusively of students from a single university, which may limit the generalizability of results to other student populations or young adults in different regions or countries. The findings may also be influenced by the characteristics of the local healthcare system and the availability of OTC medicines.

The relatively low response rate of 21.7% may have introduced non-response and selection bias. Students more interested in or experienced with self-medication may have been more likely to participate, which could have influenced the estimated prevalence and reported behaviors. As demographic data for the entire eligible student population were unavailable, comparison between respondents and non-respondents was not possible; therefore, the findings should be interpreted with caution.

Another limitation concerns the structure of the study sample. Nursing students constituted the largest subgroup and accounted for nearly two-thirds of the respondents. Therefore, the findings may primarily reflect the behaviors, attitudes, and safety-related practices of nursing students rather than those of health sciences students as a broad and internally diverse group. Although field of study was included in the logistic regression model, the relatively small number of students from some fields limited the possibility of conducting reliable field-stratified analyses. For this reason, the results should be generalized across different health-related disciplines with caution.

The important aspect to consider is the potential influence of social desirability bias on safety-related responses. Items such as following package leaflet instructions may be particularly prone to over-reporting, especially in the sample of health sciences students who are familiar with the professionally expected answers. This possibility should be taken into account when interpreting the reported safety practices.

The study design captures self-reported behaviors at a single point in time, precluding conclusions about causality or changes in self-medication practices over time. Self-reported data are also subject to recall and social desirability biases, which may have influenced responses regarding both the frequency and safety of OTC medicine use.

The item concerning adherence to package leaflet instructions showed only moderate test–retest reliability. Therefore, findings related to this safety-related practice should be interpreted with caution, as they may be less stable than results obtained for items with higher reliability coefficients.

Another aspect, contextual factors such as the characteristics of the local healthcare system, accessibility of pharmacies, and availability of OTC medicines may have influenced the observed patterns of self-medication.

The limitation of this study is also the binary classification of self-medication, which did not distinguish between occasional, low-risk use of OTC analgesics and more frequent or potentially unsafe self-medication behaviors. Consequently, routine practices (such as taking an analgesic for a headache) were grouped together with behaviors that may carry greater clinical relevance.

Another aspect to consider is that the study did not distinguish between different types of online sources used for OTC information, even though official medical websites, pharmacy portals or social media may differ in reliability. This aspect should also be kept in mind while interpreting the findings.

Despite these limitations, the study provides valuable insights into the prevalence, determinants, and safety practices of self-medication among university students, highlighting areas for targeted education and public health interventions.

## 5. Conclusions

Strengthening competencies in safe and rational self-medication should be viewed as an important public health priority, supported by continuous monitoring and refinement of preventive, educational and systemic measures. Although self-medication can, in itself, relieve the healthcare system and improve access to treatment for minor ailments, its irrational practice may pose health risks.

The increasing challenges facing the healthcare sector highlight the importance of responsible self-medication, provided individuals remain aware of both its benefits and its limitations. This is particularly important among students, who will soon become the first point of contact for many patients.

The results of the present study indicate that

- Although most students declare behaviors conducive to safety, the actual practice of conscious and responsible self-medication remains an area requiring ongoing competency improvement.
- Educational activities should focus on developing health literacy, including the critical use of medical information available online.
- Efforts should be directed toward strengthening the role of future health-related professionals as accessible patient advisors on the safe use of OTC medicines.
- Promoting rational and safe self-medication, supported by reliable knowledge and access to professional guidance, can bring meaningful benefits to both patients and the healthcare system. At the same time, gaining a clearer understanding of the factors that encourage people to self-medicate—including financial constraints, long waiting times, challenges in accessing medical appointments, or limited time for visits—is important for shaping effective public health strategies.

Taken together, these findings underscore the need for a coordinated, evidence-based approach that not only strengthens individual competencies but also addresses systemic barriers, fostering a more resilient and patient-centered model of care.

## Figures and Tables

**Table 1:** Sociodemographic and health status characteristics of respondents (*N* = 435).

Sociodemographic Factors		*n*	*%*
Age (years)	23 ± 4.2		
Gender	Female	372	85.5
	Male	61	14.0
	Other	2	0.5
Field of study	Dietetics	60	13.8
	Nursing	278	63.9
	Midwifery	22	5.1
	Emergency Medical Services	45	10.3
	Public Health	30	6.9
Mode of study	Year of study		
Full-time Bachelor’s studies	I	123	28.3
	II	26	6.0
	III	40	9.2
Full-time Master’s studies	I	66	15.2
	II	158	36.3
Part-time Bachelor’s studies	I	16	3.7
	II	4	0.9
	III	2	0.5
Place of residence	Rural	88	20.2
	Town ≤100,000	70	16.1
	City 101,000–500,000	51	11.7
	City >500,000	226	52.0
Self-assessment of financial situation	Very good	75	17.2
	Good	223	51.3
	Average	126	29.0
	Poor	8	1.8
	Very poor	3	0.7
Health Status		*N*	*%*
Self-assessment of overall health	Very good	55	12.6
	Good	265	60.9
	Average	103	23.7
	Poor	10	2.3
	Very poor	2	0.5
Presence of chronic disease	Yes	132	30.3
	No	274	63.0
	I don’t know	29	6.7

**Table 2:** Selected sociodemographic and health status factors associated with self-medication—univariable and multivariable logistic regression analysis.

Variable	Category	Self-Medication (*N* = 433)		COR (95% CI)	*p*-Value	AOR (95% CI)	*p*-Value
		Yes (*n* = 278)	No (*n* = 155)				
Sociodemographic Factors							
Age (years)	18–20	55 (19.8)	47 (30.3)	0.78 (0.29–1.07)	0.618	0.96 (0.33–2.81)	0.944
	21–23	158 (56.8)	66 (42.6)	1.59 (0.62–4.08)	0.329	1.99 (0.71–5.52)	0.188
	24–26	43 (15.5)	26 (16.8)	1.10 (0.40–3.05)	0.851	1.41 (0.47–4.23)	0.545
	27–30	10 (3.6)	8 (5.2)	0.83 (0.23–3.03)	0.782	1.16 (0.28–4.75)	0.835
	>31	12 (4.3)	8 (5.2)	1		1	
Gender	Female	255 (91.7)	117 (75.5)	3.60 (2.05–6.31)	<0.001	4.11 (2.11–8.02)	<0.001 *
	Male	23 (8.3)	38 (24.5)	1		1	
Field of study	Dietetics	36 (12.9)	24 (15.5)	0.80 (0.45–1.42)	0.445	0.94 (0.48–1.86)	0.868
	Public Health	16 (5.8)	14 (9.0)	0.61 (0.28–1.30)	0.201	0.92 (0.39–2.18)	0.846
	Emergency Medical Services	28 (10.1)	17 (11.9)	0.88 (0.46–1.70)	0.697	3.57 (1.45–8.75)	0.005 *
	Midwifery	18 (6.5)	4 (2.6)	2.40 (0.79–7.29)	0.123	2.68 (0.79–9.10)	0.115
	Nursing	180 (64.7)	96 (61.9)	1		1	
Level of study	Bachelor’s degree students	110 (39.6)	101 (65.2)	0.35 (0.23–0.53)	<0.001	0.29 (0.17–0.51)	<0.001 *
	Master’s degree students	168 (60.4)	54 (34.8)	1		1	
Place of residence	Rural	51 (18.3)	37 (23.9)	0.59 (0.35–0.98)	0.042	0.59 (0.33–1.05)	0.073
	Town ≤ 100,000	32 (11.5)	38 (24.5)	0.36 (0.21–0.62)	<0.001	0.38 (0.20–0.70)	0.002 *
	City 101,000–500,000	38 (13.7)	13 (8.4)	1.25 (0.62–2.50)	0.531	1.48 (0.68–3.19)	0.324
	City > 500,000	157 (56.5)	67 (43.2)	1		1	
Self-assessment of financial situation	Very good	54 (19.4)	21 (13.5)	2.14 (0.59–7.78)	0.247	2.10 (0.48–9.14)	0.324
	Good	142 (51.1)	81 (52.3)	1.46 (0.43–4.93)	0.542	1.40 (0.35–5.65)	0.637
	Moderate	76 (27.3)	48 (31.0)	1.32 (0.38–4.56)	0.661	1.45 (0.36–5.88)	0.606
	Poor/Very poor	6 (2.2)	5 (3.2)	1		1	
Health Status							
Self-assessment of overall health	Very good	36 (12.9)	19 (12.3)	3.80 (1.01–14.22)	0.048	1.85 (0.40–8.55)	0.432
	Good	166 (59.7)	97 (62.6)	3.42 (1.00–11.66)	0.049	1.95 (0.48–7.84)	0.348
	Fair	72 (25.9)	31 (20.0)	4.64 (1.30–16.57)	0.018	2.72 (0.67–10.99)	0.160
	Poor/Very poor	4 (1.4)	8 (5.2)	1		1	
Presence of chronic condition	Yes	80 (28.8)	50 (32.3)	0.85 (0.55–1.30)	0.449	0.72 (0.44–1.20)	0.210
	No	198 (71.2)	105 (67.7)	1		1	

Note. Data are presented as n (%). COR—crude odds ratio; AOR—adjusted odds ratio; CI—confidence interval. The reference category is indicated by OR = 1.00. AORs were estimated using a multivariable logistic regression model with the forced-entry method and were adjusted for age, gender, field of study, level of study, place of residence, self-assessed financial situation, self-assessed overall health, and presence of chronic condition. Model fit statistics were as follows: χ^2^(20) = 75.440, *p* < 0.001; −2 log likelihood = 489.399; Cox and Snell R^2^ = 0.160; Nagelkerke R^2^ = 0.219; Hosmer–Lemeshow test: χ^2^(8) = 6.649, *p* = 0.575; overall classification accuracy = 69.5%. Multicollinearity diagnostics indicated no evidence of problematic multicollinearity, with tolerance values ≥ 0.785 and VIF values ≤ 1.274. * *p* < 0.05.

**Table 3:** Self-medication behaviors among students using OTC medicines (N = 278).

Aspect	Response	*n*	*%*
(I) Attitudes toward SM			
Reasons for SM	Lack of time for a medical visit	141	50.7
	Easy access to medications	218	78.4
	Lack of financial resources for prescribed medications	10	3.6
	Long queues at outpatient clinics	70	25.2
	Long waiting time for a doctor’s appointment	107	38.5
	Previous dissatisfaction with medical services	22	7.9
	Lack of trust in physicians	9	3.2
	High effectiveness and quick therapeutic effect	128	46.0
	Media advertisements	0	0
Perceived advantages of SM	Saving time	101	36.3
	Engagement and self-determination in one’s own health	69	24.8
	Relieving doctors and increasing the time available for treating more serious diseases	58	20.9
	Saving money	11	4.0
	Reduction of healthcare system costs	5	1.8
	No noticeable advantages	37	13.3
Perceived risks of SM	Underestimation of symptoms of a serious diseases	191	68.7
	Incorrect self-diagnosis	159	57.2
	Adverse interactions due to improper combination of multiple medications	132	47.5
	Delayed diagnosis of disease	111	39.9
	Unnecessary use of medicines	111	39.9
	Risk of medication dependence	57	20.5
(II) Behaviors related to purchasing and using OTC medicines			
Types of OTC medicines used/Health purpose	Analgesics	271	97.5
	Vitamins	152	54.7
	Antipyretics	134	48.2
	Antispasmodics	77	27.7
	Antitussives	60	21.6
	Immune-supporting preparations	48	17.3
	Sedatives and sleep aids	36	12.9
	Antiallergic medications	29	10.4
	Memory and concentration enhancers	10	3.6
Place of purchase of OTC medicines	Pharmacy	257	92.4
	Grocery store, supermarket	12	4.3
	Beauty supply store	6	2.2
	Gas station	0	0
	Newspaperstand	0	0
	Internet	3	1.1
Frequency of OTC medicine purchase	Several times a month	21	7.6
	Once a month	79	28.4
	Several times a year	170	61.2
	Once a year	8	2.9
Duration of SM	2–3 days	105	37.8
	4–7 days	129	46.4
	8–10 days	4	1.4
	Until improvement occurs	40	14.4
Sources of information about OTC medicines	Internet	184	66.6
	Pharmacist	159	57.2
	Family	132	47.5
	Physician	131	47.1
	Friends	59	21.2
	Advertisements	0	0.0
(III) Safety-related practices			
Adherence to the medicine leaflet instructions	Yes, always	121	43.5
	Almost always	127	45.7
	Sometimes	22	7.9
	Only partially	6	2.2
	Never	0	0
	I do not read leaflets	2	0.7
Use of medicines beyond their expiration date	Yes, often	8	2.9
	Sometimes	55	19.8
	Never	215	77.3

**Table 4:** Summary of associations between sociodemographic and health factors and self-medication behaviors from the area of attitudes (Chi-square test).

Health Behaviors	Sociodemographic Factors and Health Status	Association	FDR
(I) Attitudes toward SM			
Reasons for SM (yes/no): Lack of time for a medical visit Easy access to medications Lack of financial resources for prescribed medications Long queues at outpatient clinics Long waiting time for a doctor’s appointment Previous dissatisfaction with medical services Lack of trust in physicians High effectiveness and quick therapeutic effect Media advertisements	Age (years)	Students aged > 31 years ↑ lack of time for a medical visit as a reason for SM (*p* = 0.040; *V* = 0.190)	0.158
		Students aged 18–20 and 24–26 years ↑ lack of financial resources for prescribed medications as a reason for SM (*p* = 0.009; *V* = 0.222)	0.074
		Students aged > 31 years ↑ previous dissatisfaction with medical services as a reason for SM (*p* = 0.001; *V* = 0.254)	0.014 **
	Gender *	Women ↑ tolerance of long waiting time as a reason for SM (*p* = 0.009; *V* = 0.157)	0.074
	Field of study	Emergency Medical Services and Nursing students ↑ long queues at outpatient clinics as a reason for SM (*p* = 0.005; *V* = 0.233)	0.051
		Midwifery students ↑ previous dissatisfaction with medical services as a reason for SM (*p* < 0.001; *V* = 0.273)	0.014 **
	Level of study	Bachelor’s degree students ↑ lack of financial resources for prescribed medications as a reason for SM (*p* = 0.008; *V* = 0.160)	0.069
	Place of residence	Large-city residence ↑ lack of time for a visit as a reason for SM (*p* = 0.015; *V* = 0.194)	0.101
		Large-city residence ↑ long waiting queues as a reason for SM (*p* = 0.016; *V* = 0.193)	0.101
	Self-assessment of financial situation	Moderate financial status ↑ financial barriers to prescribed treatment (*p* < 0.001*; *V* = 0.315) Good financial status ↑ long waiting time as a reason for SM (*p* = 0.018; *V* = 0.202)	0.014 **
			0.107
	Presence of chronic disease	Chronic disease ↑ dissatisfaction with healthcare services as a reason for SM (*p* < 0.001; *V* = 0.229)	0.014 **
	Self-assessment of overall health	Fair health status ↑ lack of trust in physicians (*p* = 0.039; *V* = 0.174) Good/very good health ↑ perceived effectiveness and rapid effect of SM (*p* = 0.014; *V* = 0.195)	0.158
			0.101
Perceived advantages of SM (yes/no): Saving time Engagement and self-determination in one’s own health Relieving doctors and increasing the time available for treating more serious diseases Saving money Reduction in healthcare system costs No noticeable advantages	Age (years)	Students aged 27–30 years ↑ saving time as an advantage of SM (*p* = 0.014; *V* = 0.211)	0.101
	Gender *	Women ↑ engagement and self-determination in one’s own health (*p* = 0.031; *V* = 0.130)	0.139
	Field of study	Public Health, Midwifery and Emergency Medical Services students ↑ saving money as an advantage of SM (*p* = 0.019; *V* = 0.206)	0.107
		Emergency Medical Services and Public Health students ↑ saving time as an advantage of SM (*p* = 0.040; *V* = 0.190)	0.158
		Midwifery students ↑ reduction in healthcare system costs as an advantage of SM (*p* = 0.029; *V* = 0.197)	0.136
	Level of study	ns	-
	Place of residence	Residents of smaller towns ↑ reporting saving money as an advantage of SM (*p* < 0.001; *V* = 0.254) Residents of large cities ↑ reporting saving time as the main advantage of SM (*p* = 0.039; *V* = 0.174)	0.014 **
			0.158
	Self-assessment of financial situation	ns	-
	Presence of chronic disease	Individuals with chronic diseases ↑ indicating relieving physicians as an advantage of SM (*p* = 0.018; *V* = 0.170)	0.107
	Self-assessment of overall health	ns	-
Perceived risks of SM (yes/no): Underestimation of symptoms of a serious diseases Incorrect self-diagnosis Adverse interactions due to improper combination of multiple drugs Delayed diagnosis of disease Unnecessary use of medicines Risk of medication dependence	Age (years)	• Students aged 18–23 years ↑ risk of medication dependence as a perceived risk of SM (*p* = 0.015; *V* = 0.211)	0.101
	Gender *	Women ↑ indicating risks related to potential drug interactions (*p* = 0.002; *V* = 0.180) Men ↑ indicating underestimation of disease symptoms (*p* = 0.014)	0.024 **
			0.101
	Field of study	Emergency Medical Services and Public Health students ↑ underestimation of symptoms of serious disease as a perceived risk of SM (*p* = 0.002; *V* = 0.244)	0.024 **
		Public Health and Dietetics students ↑ delayed diagnosis of disease as a perceived risk of SM (*p* = 0.010; *V* = 0.219)	0.080
		Nursing and Midwifery students ↑ risk of medication dependence as a perceived risk of SM (*p* = 0.039; *V* = 0.190)	0.158
	Level of study	ns	-
	Place of residence	Large-city residence ↑ underestimation of symptoms (*p* < 0.001; *V* = 0.255) Large-city residence ↑ incorrect self-diagnosis (*p* = 0.042; *V* = 0.172)	0.014 **
			0.163
	Self-assessment of financial situation	Good financial status ↑ delayed diagnosis (*p* = 0.020; *V* = 0.188) Good financial status ↑ unnecessary use of medicines (*p* = 0.016; *V* = 0.192) Good financial status ↑ risk of medication dependence (*p* = 0.010; *V* = 0.202)	0.109
			0.101
			0.080
	Presence of chronic disease	Chronic disease ↑ unnecessary use of OTC medicines (*p* = 0.021; *V* = 0.167)	0.110
	Self-assessment of overall health	Fair health status ↑ risk of medication dependence (*p* < 0.001; *V* = 0.243) Fair health status ↑ risk of drug interactions (*p* = 0.002; *V* = 0.228)	0.014 **
			0.024 **

↑ more frequent; ↓ less frequent; ns—not statistically significant in the chi-square test; *p*—nominal *p*-value from the chi-square test; FDR—Benjamini–Hochberg FDR-adjusted *p*-value; *V*—Cramer’s *V*. * Respondents who selected “Other” for gender were excluded from gender-based analyses ** Statistically significant after FDR correction (FDR < 0.05).

**Table 5:** Summary of associations between sociodemographic and health factors and self-medication behaviors from the area of purchase and use of OTC medicines (Chi-square test).

Health Behaviors	Sociodemographic Factors and Health Status	Association	FDR
(II) Practices related to the purchase and use of OTC medicines			
Types of OTC medicines used/Health purpose (yes/no): Symptomatic medicines Analgesics Antipyretics Antispasmodics Antitussives Health-supporting medicines Vitamins Immune-supporting preparations Sedatives and sleep aids Antiallergic medications Memory and concentration enhancers Others	Age (years)	• Students aged 18–20 years ↑ use of antispasmodics (*p* = 0.043; *V* = 0.188)	0.163
		• Students aged > 31 years ↑ use of vitamins (*p* = 0.032; *V* = 0.195)	0.141
		• Students aged 18–20 years ↑ use of sedatives and sleep aids (*p* = 0.019; *V* = 0.206)	0.107
	Gender *	Women ↑ use of antispasmodics (*p* = 0.002; *V* = 0.186)	0.024 **
	Field of study	• Nursing students ↑ use of antipyretics (*p* = 0.005; *V* = 0.232)	-
		• Midwifery and dietetics students ↑ use of antispasmodics (*p* = 0.026; *V* = 0.200)	0.128
	Level of study	• Master’s students ↑ use of antipyretics (*p* < 0.001; *V* = 0.251)	0.014 **
		• Bachelor’s students ↑ use of sedatives and sleep aids (*p* < 0.001; *V* = 0.214)	0.014 **
	Place of residence	Large-city residence ↑ use of antitussives (*p* = 0.035; *V* = 0.176) Medium/large-city residence ↑ use of sedatives and sleep aids (*p* = 0.027; *V* = 0.182)	0.150
			0.128
	Self-assessment of financial situation	Good financial status ↑ use of immune-supporting preparations (*p* = 0.005; *V* = 0.214) Only respondents with good financial status ↑ use of memory-enhancing preparations (*p* = 0.019; *V* = 0.189)	0.051
			0.107
	Presence of chronic disease	Chronic disease ↑ use of analgesics (*p* < 0.001; *V* = 0.238) Absence of chronic disease ↑ use of antitussives (*p* = 0.001; *V* = 0.217)	0.014 **
			0.014 **
	Self-assessment of overall health	ns	-
Sources of information about OTC medicines (yes/no): Internet Pharmacist Family Physician Friends Advertisements	Age (years)	• Students aged 18–20 years ↑ using the Internet as a source of information about OTC medicines (*p* = 0.003; *V* = 0.238)	0.033 **
		• Students aged 27–30 years ↑ consulting family members as a source of information about OTC medicines (*p* < 0.001; *V* = 0.329)	0.014 **
	Gender *	Men ↓ consulting a physician as a source of information about OTC medicines (*p* = 0.003; *V* = 0.179)	0.033 **
	Field of study	Dietetics students ↑ consulting a physician as a source of information about OTC medicines (*p* = 0.011; *V* = 0.217)	0.084
		• Public Health students ↑ using the Internet as a source of information about OTC medicines (*p* = 0.011; *V* = 0.217)	0.084
		Midwifery students ↑ consulting family members as a source of information about OTC medicines (*p* < 0.001; *V* = 0.276)	0.014 **
	Level of study	• Master’s students ↑ consulting a physician as a source of information about OTC medicines (*p* = 0.008; *V* = 0.160)	0.069
		• Master’s students ↑ using the Internet as a source of information about OTC medicines (*p* = 0.001; *V* = 0.199)	0.014 **
		Bachelor’s students ↑ consulting family members as a source of information about OTC medicines (*p* < 0.001; *V* = 0.291)	0.014 **
	Place of residence	Large-city residence ↑ consulting a pharmacist (*p* = 0.027; *V* = 0.182) Small-town and rural residence ↑ consulting friends (*p* = 0.001; *V* = 0.248) Small-town and rural residence ↑ consulting family members (*p* = 0.045; *V* = 0.170)	0.128
			0.014 **
			0.169
	Self-assessment of financial situation	Good and very good financial status ↑ consulting a physician (*p* < 0.001; *V* = 0.272) Moderate financial status ↓ consulting a physician (*p* < 0.001; *V* = 0.272)	0.014 **
			0.014 **
	Presence of chronic disease	Chronic disease ↑ consulting a pharmacist (*p* = 0.002; *V* = 0.216) Chronic disease ↑ using the Internet (*p* = 0.042; *V* = 0.151) Chronic disease ↑ consulting friends (*p* = 0.030; *V* = 0.158) Chronic disease ↑ consulting family (*p* = 0.047; *V* = 0.148)	0.024 **
			0.163
			0.138
			0.170
	Self-assessment of overall health	Good/very good health ↑ consulting friends as a source of medication advice (*p* = 0.020; *V* = 0.188)	0.109

↑ more frequent; ↓ less frequent; ns—not statistically significant in the chi-square test; *p*—nominal *p*-value from the chi-square test; FDR—Benjamini–Hochberg FDR-adjusted *p*-value; *V*—Cramer’s *V*. * Respondents who selected “Other” for gender were excluded from gender-based analyses. ** Statistically significant after FDR correction (FDR < 0.05). Note. Place of purchase, frequency of OTC medicine purchase, and duration of self-medication were analyzed descriptively only. Place of purchase was excluded from association analyses due to the highly uneven distribution of responses, with most respondents indicating pharmacies (92.4%) and some categories having zero counts. Frequency of purchase and duration of self-medication were not primary outcomes in this part of the analysis.

**Table 6:** Summary of associations between sociodemographic and health factors and selected self-medication behaviors from the area of safety-related practices (Chi-square test).

Health Behaviors	Sociodemographic Factors and Health Status	Association	FDR
(III) Safety-related practices			
Adherence to the medication leaflet instructions (always, almost always/sometimes, to some extent):	Age (years)	Students aged 18–20 years ↑ adherence to medication leaflet instructions (*p* = 0.008; *V* = 0.225)	0.069
	Gender *	Women ↑ adherence to leaflet instructions (*p* = 0.008; *V* = 0.225)	0.069
	Field of study	Dietetics and nursing students ↑ adherence to medication leaflet instructions (*p* = 0.016; *V* = 0.210)	0.101
	Level of study	ns	-
	Place of residence	Large-city residence ↑ correct adherence to leaflet instructions (*p* = 0.003; *V* = 0.190)	0.033 **
	Self-assessment of financial situation	ns	-
	Presence of chronic disease	Chronic disease ↑ adherence to leaflet instructions (*p* = 0.021; *V* = 0.168)	0.110
	Self-assessment of overall health	ns	-
Use of medications beyond their expiration date (yes/sometimes/no):	Age (years)	Students aged 27–30 years and >31 years ↑ use of medications beyond their expiration date *(p* < 0.001; *V* = 0.274).	0.014 **
	Gender *	ns	-
	Field of study	Public Health, Midwifery, and Emergency Medical Services students ↑ use of medications beyond their expiration date (*p* = 0.008; *V* = 0.193)	0.069
	Level of study	Bachelor’s students ↑ use of medications beyond their expiration date (*p* = 0.006; *V* = 0.191)	0.061
	Place of residence	ns	-
	Self-assessment of financial situation	ns	-
	Presence of chronic disease	ns	-
	Self-assessment of overall health	ns	-

↑ more frequent; ↓ less frequent; ns—not statistically significant in the chi-square test; *p*—nominal p-value from the chi-square test; FDR—Benjamini–Hochberg FDR-adjusted *p*-value; *V*—Cramer’s *V*. * Respondents who selected “Other” for gender were excluded from gender-based analyses. ** Statistically significant after FDR correction (FDR < 0.05).

## Data Availability

The dataset generated and/or analyzed during the current study is available from the corresponding author on reasonable request. The dataset is not publicly available due to privacy and ethical considerations related to the anonymous survey data and the conditions under which participants provided their responses.

## Supplementary Materials

The following supporting information can be downloaded at: https://www.mdpi.com/article/10.3390/healthcare14131910/s1, File S1: Rational and safe self-medication—A survey addressed to students of the Faculty of Health Sciences at the Medical University of Warsaw.

## Abbreviations

The following abbreviations are used in this manuscript:

SM	Self-medication
OTC	Over-the-counter
WHO	World Health Organization
COR	Crude odds ratios
CI	Confidence Interval
SD	Standard Deviation
