# Supplementary material for: Self-Medication Behaviors: Determinants, Motivations, and Safety Practices Among Health Sciences Students
Source: Healthcare (Basel). 2026 Jul 1;14(13):1910. doi: 10.3390/healthcare14131910 (PMC13362289; doi:10.3390/healthcare14131910)
Supplement: Supplementary file 1 [file healthcare-14-01910-s001.zip › healthcare-4363867-supplementary.pdf]

File S1

Rational and safe self-medication – a survey addressed to students of the Faculty of Health Sciences at the Medical University of Warsaw

We invite you to take part in a survey aimed at assessing awareness and behaviours relating to self-medication among future healthcare professionals.

The survey is completely anonymous and its results will be used solely for research purposes.

Self-medication refers to as the practice of using medicinal products to treat self-diagnosed disorders or symptoms, as well as the periodic or regular use of previously prescribed medicines for chronic or recurrent conditions or symptoms.

The socio-demographic section

1. Please select your gender:
   a. Female
   b. Male
   c. Other

2. Please enter your age (in years): ……………..

3. What is your field of study?
   a. Dietetics
   b. Nursing
   c. Midwifery
   d. Emergency Medical Services
   e. Public Health

4. What is your mode of study?
   a. Full-time Bachelor's studies
   b. Full-time Master's studies
   c. Part-time Bachelor's studies

5. Please enter your year of study:
   a. I
   b. II
   c. III

6. Where do you live?
    a. Rural
    b. Town ≤100,000
    c. City 101,000–500,000
    d. City >500,000

a. How would you rate your financial situation?
    a. Very good
    b. Good
    c. Average
    d. Poor
    e. Very poor

7. How would you rate your overall health?
    a. Very good
    b. Good
    c. Average
    d. Poor
    e. Very poor

8. Do you have a chronic illness?
    a. Yes
    b. No
    c. I don't know

The section on self-medication.

9. Have you used over-the-counter medicines in the past 12 months, for example, when you experienced mild health ailments (such as a mild sore throat or a canker sore)?
    a. Yes
    b. No

10. Which over-the-counter medications do you use on your own most often? (You may select more than one answer)
    a. Analgesics
    b. Vitamins
    c. Antipyretics
    d. Antispasmodics
    e. Antitussives
    f. Immune-supporting preparations

g. Sedatives and sleep aids
h. Antiallergic medications
i. Memory and concentration enhancers

11. Where do you typically purchase over-the-counter medicines?
    a. Pharmacy
    b. Grocery store, supermarket
    c. Beauty supply store
    d. Gas station
    e. Newspaperstand
    f. Internet

12. How often do you purchase over-the-counter medicines?
    a. Several times a month
    b. Once a month
    c. Several times a year
    d. Once a year or less

13. When you use over-the-counter medicines, what is the typical duration of treatment?
    a. 2-3 days
    b. 4-7 days
    c. 8-10 days
    d. Until improvement occurs

14. What are your reasons for using over-the-counter medicines? (You may select more than one answer)
    a. Lack of time for a medical visit
    b. Easy access to medications
    c. Lack of financial resources for prescribed medications
    d. Long queues at outpatient clinics
    e. Long waiting time for a doctor's appointment
    f. Previous dissatisfaction with medical services
    g. Lack of trust in physicians
    h. High effectiveness and quick therapeutic effect
    i. Media advertisements

15. Which source do you use to find information about over-the-counter medicines? (You may select more than one answer)
    a. Pharmacist
    b. Physician
    c. Family

d. Friends
e. Internet
f. Advertisements

16. In your opinion, what are the greatest advantages of self-treatment?
    a. Saving time
    b. Engagement and self-determination in one's own health
    c. Relieving doctors and increasing the time available for treating more serious diseases
    d. Saving money
    e. Reduction of healthcare system costs
    f. No noticeable advantages

17. In your opinion, what are the main risks associated with self-medication?
    a. Underestimation of symptoms of a serious diseases
    b. Incorrect self-diagnosis
    c. Adverse interactions due to improper combination of multiple medications
    d. Delayed diagnosis of disease
    e. Unnecessary use of medicines
    f. Risk of medication dependence

18. Do you follow the information provided in the package leaflet for over-the-counter medicines?
    a. Yes, always
    b. Almost always
    c. Sometimes
    d. Only partially
    e. Never
    f. I do not read leaflets

19. Do you take any over-the-counter medicines that have passed their expiration date?
    a. Yes, often
    b. Sometimes
    c. Never

Thank you for completing the questionnaire!
